# Virtual Reality Based Support System for Layout Planning and Programming of an Industrial Robotic Work Cell

**DOI:** 10.1371/journal.pone.0109692

**Published:** 2014-10-31

**Authors:** Hwa Jen Yap, Zahari Taha, Siti Zawiah Md Dawal, Siow-Wee Chang

**Affiliations:** 1 Department of Mechanical Engineering, Faculty of Engineering, University of Malaya, Kuala Lumpur, Malaysia; 2 Faculty of Mechanical Engineering, Universiti Malaysia Pahang, Pekan, Pahang, Malaysia; 3 Bioinformatics Program, Institute of Biological Sciences, Faculty of Science, University of Malaya, Kuala Lumpur, Malaysia; ICREA-University of Barcelona, Spain

## Abstract

Traditional robotic work cell design and programming are considered inefficient and outdated in current industrial and market demands. In this research, virtual reality (VR) technology is used to improve human-robot interface, whereby complicated commands or programming knowledge is not required. The proposed solution, known as VR-based Programming of a Robotic Work Cell (VR-Rocell), consists of two sub-programmes, which are VR-Robotic Work Cell Layout (VR-RoWL) and VR-based Robot Teaching System (VR-RoT). VR-RoWL is developed to assign the layout design for an industrial robotic work cell, whereby VR-RoT is developed to overcome safety issues and lack of trained personnel in robot programming. Simple and user-friendly interfaces are designed for inexperienced users to generate robot commands without damaging the robot or interrupting the production line. The user is able to attempt numerous times to attain an optimum solution. A case study is conducted in the Robotics Laboratory to assemble an electronics casing and it is found that the output models are compatible with commercial software without loss of information. Furthermore, the generated KUKA commands are workable when loaded into a commercial simulator. The operation of the actual robotic work cell shows that the errors may be due to the dynamics of the KUKA robot rather than the accuracy of the generated programme. Therefore, it is concluded that the virtual reality based solution approach can be implemented in an industrial robotic work cell.

## Introduction

According to a survey from the UN Economic Commission for Europe published in “World Robotics 2001” [1], there were approximately 750,000 robots running in industries worldwide. After a decade, the statistics, market analysis, forecasts, case studies and profitability of robot investment published by the International Federation of Robotics (IFR) in “World Robotics 2011” [2] revealed that the annual robot sales in 2010 was 118,337 units. The sales almost doubled compared to that in 2009, during the worldwide economic and financial crisis. The forecasted figures for year 2011 and 2014 were 139,300 and 166,700 respectively. On the other hand, the sale for robots in 2010 was US$ 5.7 billion. It was estimated that the sales value for robot systems in 2010 was US$ 17.5 billion, which includes software, peripherals and systems engineering. On the other hand, over 1,300 labours were struck by moving objects in their workplace. This appears to be the most common accident which causes injuries, which is due to the complexity of hardware and software. In Sweden and Japan [3], these incidents have been associated with robot operations, such as malfunction to stop, rapid motion of the robot arm, injuries in the workspace during repair and maintenance, illegal entrance to the workspace as well as unpredictable robot work functions to the operator.

In this research, a virtual reality based programming of a robotic work cell (VR-Rocell) is proposed and developed. In general, robot programming methods can be classified as online and offline programming, which is dependent on the presence of the actual robot while the programme is being created. In online programming, the actual robot must be present and used in the teaching process. Offline programming is the most popular method for robot programming as it does not involve real robots. This reduces robot downtime and the production line will be unaffected [4]. The system is designed to improve human-machine interface and is divided into two major sub-systems, namely, VR-Robotic Work Cell Layout (VR-RoWL) and VR-Robot Teaching System (VR-RoT). Open platform architecture is used along with a universal file format. The system uses the teach-points concept for offline programming to generate robot commands, which prevents users from potential uncertain manufacturing environments. The objective of this research is to develop a VR-based system for cell layout planning and design, path teaching, programming and simulation for an industrial robot which will achieve zero robot downtime during path teaching using a generic approach.

## Solution Approaches for an Industrial Robotic Work Cell

The synergy between virtual reality and robotics in various areas was reviewed in 1999 [5]. It is known that traditional approaches in robot programming are tedious and require knowledge of a specific robot language. Hence, a new virtual reality based system is needed to provide users with a more user-friendly interface by means of high-level programming language. Various solution approaches for industrial robots have been proposed in past studies and are presented in the following sections.

### Simulation Solution Approaches

There are various commercial software available which provide solutions for facility layout planning involving robotic work cells. In addition, commercial software have also been specifically developed for industrial robot simulation and visualization. Simulation and visualization of robotic work cells are necessary prior to procurement of hardware. The simulation software allow users to check the reachability, workspace, safety issues as well as other aspects of industrial robots.

In general, facility layout planning software is based on the discrete-event simulation concept, in which the operation of the system corresponds accordingly to the chronological order of events. Discrete-event simulation software provides an overall picture of the simulated robotic work cell, whereby the industrial robot is considered as an event within the cell. Hence, detailed simulation and information of the industrial robot (such as path planning and robot programming) may be unavailable in this type of simulation since robot commands cannot be generated automatically from the simulation results. In addition, the simulation environment does not consider spatial constraints and ergonomic issues. Although this technique is most popular compared to other techniques (e.g. gaming), it has a relatively low stakeholder commitment due to modelling lead time and purpose [6].

Geometric or continuous simulation provides a geometric graphical representation with constant time interval. Unlike discrete-event simulation with irregular time intervals, geometric simulation is more suitable for 3D visualization, offline programming of robots and collision detection during manufacturing processes [7]. Geometric simulation is also called virtual manufacturing [8].

On the other hand, various robotic simulation software have been developed by robot manufacturers such as ABB, KUKA, Fanuc and Mitsubishi. In this research, industrial robots manufactured by KUKA are used. KUKA SimPro is the official software developed specifically for offline programming of KUKA robots. The product is connected to KUKA OfficeLite, which is a virtual KUKA controller and enables real-time cycle time analyses and generation of robot programmes. However, the software requires adequate knowledge of robotic systems and special training. The cost of the software may not be within the financial means of many industries.

There are several generic robotic software available in the market such as Delmia from Dassault Systems, RobCAD from Technomatrix, Robsim from Camelot and Cosimir from Festo. These packages are flexible for various industrial robots produced by different manufacturers and are fully coupled with product life management (PLM). However, the disadvantages of these simulation software are the absence of direct outputs to the actual robots and require strong knowledge of robotic systems. The fundamental criteria which enable the adoption of such software in SMEs and academic sectors are affordability, ease of use and short learning curves [9], in which generic robot software are unable to do so. Furthermore, system integration is particularly a challenging task, whereby compatibility issues between some robot manufacturers and data structure of different software vendors may occur. Thus, simulation software with open architecture are actively developed by many researchers, in which the codes written for the simulation are portable to real robotic platforms [10].

### Virtual Manufacturing Solution Approaches

Virtual Manufacturing (VM) is the use of virtual reality technology in real-time manufacturing based simulations in order to optimize product design and processes for a specific manufacturing goal such as design for assembly, quality, lean operations, and/or flexibility. VM is used to generate 3D models and real-time simulations of manufacturing processes to aid the design and production of products. VM is known as the next revolution of global manufacturing [11].

Lawrence Associates [12] introduced three virtual manufacturing paradigms which have been applied by Kim & Choi [13] to solve problems in flexible manufacturing systems. These three paradigms are:

- Design-centred VM: Provides designers/engineers with tools to design products which fulfil the design criteria. Design-centred VM can be performed early during the conceptual design stage, which is easily modified and tested. This type of VM also uses simulation technology to optimize design specifications such as stress, strain and failure analyses.
- Production-centred VM: Provides the means to develop and analyse alternative production and process plans. Factory models and layout designs can be created to simulate the production process, assembly process, material flow and human factors. Production simulation is capable of simulating manufacturing process models which provides a means for inexpensive, fast evaluation of various processing alternatives.
- Control-centred VM: Evaluates product designs, production plans and control strategies. This VM interactively improves these three aspects through simulations of the control process. It is an additional tool to control models and actual processes, enabling seamless simulation for optimization during the actual production cycle.

Mujber et al. [14] classified the applications of VR in manufacturing into three areas, which are design, operations management and manufacturing process. In the design area, the VM technique has been used to design the die geometry for sheet metal forming in a virtual space. It is also widely used to generate virtual prototypes in order to replace physical prototypes [15]. The time required for design and development can be shortened and costs can be reduced when producing physical prototypes. Furthermore, virtual prototypes can be used to support design and development through simulation and analysis results [16], [17]. The integration of virtual reality with rapid prototyping technology is capable of creating digital prototypes to assist product development.

Moreover, VM technology is widely employed in the planning phase of manufacturing processes. This technology is used to estimate, optimize and minimize the processing time prior to the actual production [18]. In addition, this technology can be used to investigate material and information flow through the virtual operation control concept. The factors affecting the operation of a manufacturing system can be tracked to evaluate design and operational performance. In-house virtual training can be developed for machine tools [19], milling machines [20], turn-milling centres [21], maintenance [22] and so on.

VR application in manufacturing processes covers machining, assembly and inspection. Virtual machining is commonly used to solve cutting processes such as milling, turning and drilling. Collision detection can be checked to prevent accidental collisions which will damage machine tools during actual processes [23]. In addition, VM can be used in assembly planning to investigate the feasibility of assembly processes. The mechanical and physical characteristics of equipment and tooling can be modelled and simulated virtually. Furthermore, the quality, quantity, product cycle and cost can be determined in order to obtain optimum results. Virtual inspection is used to check the physical and mechanical properties of inspection equipment such as collision checks [24].

The effectiveness of VR-based training systems has also been tested. A VR-based training system for Metal Active Gas Welding (MAG) has been developed to integrate virtual robot path teaching system with a practical training system, in which VR-based offline robot programming techniques are implemented. The process paths are triggered, recorded and converted into robot commands for robotic welding. A survey has been carried out on controlled and uncontrolled groups of students in Faculty of Engineering, University of Malaya, in order to evaluate their motivation towards virtual welding learning and conventional welding learning. The results revealed that more than 85% of the students supported the use of VR integrated training system compared to traditional welding training. The respondents agreed that VR technology is a valuable tool and the prototype with VR-based system is feasible to be implemented as a supplement in the welding training programme as well as in path planning for robotic welding [4].

## Materials and Methods

Due to limited accessibility, high cost and strong demand for technical knowledge for commercial simulation software, a number of research projects have been conducted to develop alternative solution approaches for industrial robots. Several virtual robots have been created in the works of [25]–[27]. However, these systems still require strong technical knowledge. Furthermore, the interactions between humans and virtual robots are very poor, and are limited to conventional input/output devices.

VR-based industrial robotic systems are able to overcome the above mentioned problems [28]. VR technology was used to develop a virtual gripper for robotic inspections such that information of the inspected workpiece (position and orientation) can be defined interactively. The synergy between virtual reality and robots was reviewed and it was concluded that VR in robotics can be attained from haptic interfaces and human factor know-how [5]. Xu et al. [29] developed VR-based robot graphics simulation with built-in collision detection and coordinate translation functions.

Santis and Siciliano [30] developed a physical human-robot interaction model to measure the safety and dependability of robots. This model was implemented to evaluate the perceived safety during robot motion, which is dependent on shape, speed and posture. VR technology was also used to model a complex robotics system to study the perception of safe robot idle time [31]. They found that robot speed and size has an effect on idle time, whereby high robot speed and large-sized robot increases the waiting time of the participants.

**Figure 1:**
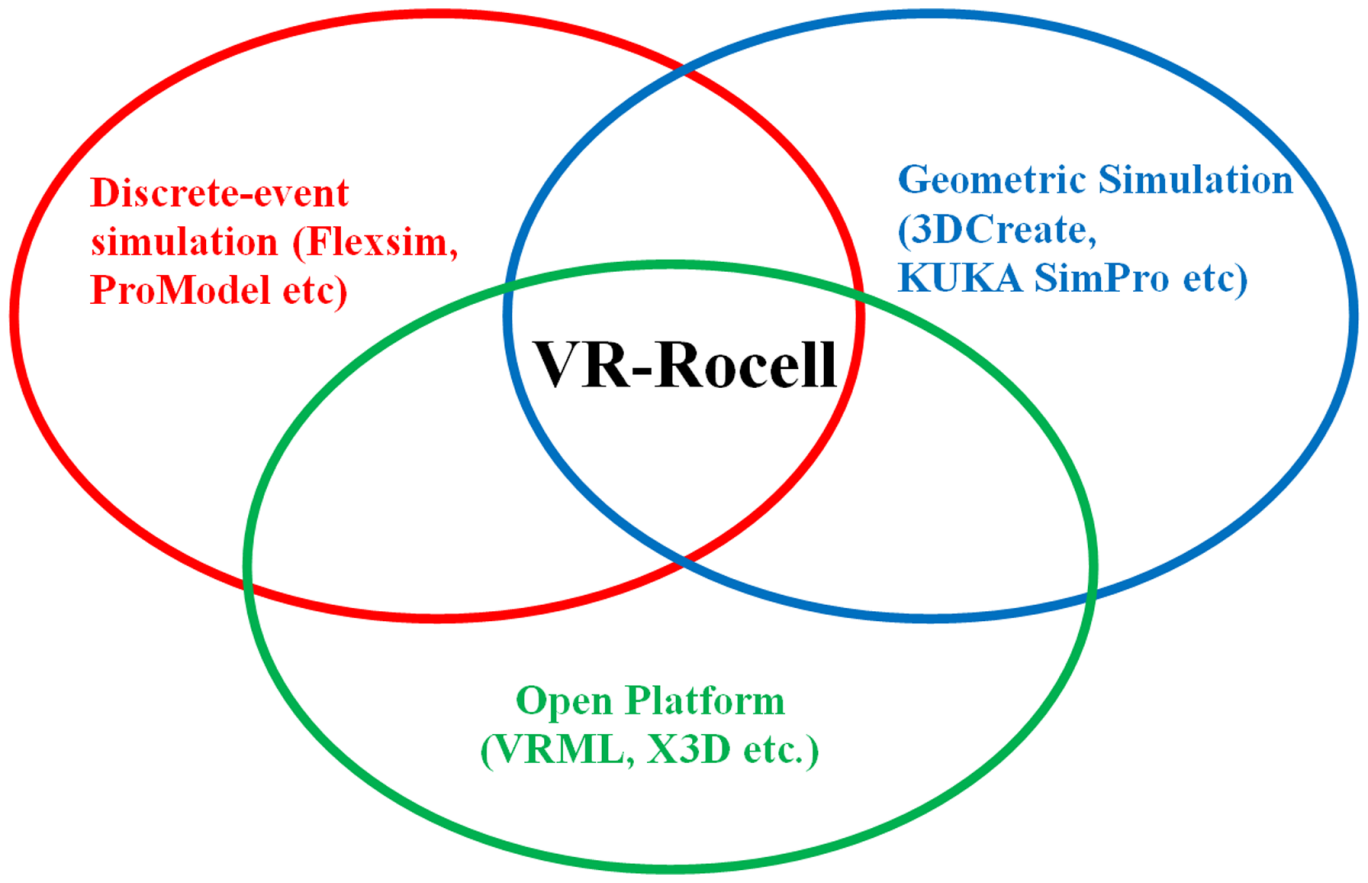
Proposed model of VR-Rocell.

Figure 1 shows the proposed model of this research, which is VR-Rocell. The model is designed to integrate the advantages of various simulation systems into a robotic work cell. The model proposes a VR-based layout planning and design for robotic work cell, which is similar to the functions provided by discrete-event simulation software. Furthermore, robot programming is conducted based on the teach-by-demonstrate concept for the VR-based robot teaching system. Robot paths are taught through an intuitive user interface compared to geometric simulation systems. The proposed model is based on open platform architecture, which includes programming language (C++), graphic libraries (OpenGL) and 3D formats (e.g. VRML).

CAD format can be converted into virtual reality (VR) by using either a commercial modelling software, library or database approach [32]. 3D models can be integrated in virtual manufacturing (VM) through Lex lexical analyser generator and Open Graphic Library [33]. However, there are no standard methods or algorithms which convert 3D models from CAD modelling software into VR and/or VM, which is monitored and controlled by the related society. In addition, there are no standard methods or algorithms which convert 3D models from CAD modelling software to virtual manufacturing systems. Therefore, a generic algorithm is proposed to import 3D models into the VR-based system based on an open platform concept. The system can be used in all operating systems such as Windows, Mac and Linux. In addition, the system supports all types of browsers such as Internet Explorer, Mozilla and Google Chrome.

Most of the commercial VR packages use native file formats to create their virtual environment. These formats consist of algorithms and data structures for a particular software, which may not be readable or visualized by others. The universal formats which are loaded into the CAD software are not editable in the native system. The assembly of universal formats cannot be saved back into the universal format. For these reasons, the proposed model consists of four universal file formats, which are supported by all related software. An algorithm is developed to extract and save 3D meshing information into four file formats. File format conversion is possible among the above formats. Thus, the output models from one VM can be transferred or imported to another VM using a universal file format. A database can be built to re-configure a modular-based VM, which further reduces the development time of the new system [33], [34]. The developed VR-based system is designed with simple and user-friendly interfaces to reduce the software training period and is therefore suitable for inexperienced users. Furthermore, the system is created based on an open architecture concept which allows the generated data to be transferred to/from different platforms without loss of information. In this manner, the output data can be viewed and configured by other systems.

The issues on human-computer interface design parameters in virtual environment have also been studied. A systematic axiomatic design method was used to design the virtual environment in order to minimize visual symptoms. The axiomatic design parameters were identified such as colour of background, brightness of virtual lighting, field of view (FOV), speed of virtual objects and display resolution. This method was developed and tested on a virtual robot manufacturing system [35].

### Hardware Architecture

In this research, the hardware of the system is divided into two major parts, which are computer workstation and virtual reality hardware. The computer workstation consists of the computer CPU with a dedicated graphics card and is connected to two monitors, video splitters, two projectors, display extension devices and other peripherals (e.g. mouse, keyboard and speaker).

A Virtual Wall system has been built for the virtual reality hardware. The frame is made of an aluminium profile (40 mm×40 mm), which can be easily assembled and disassembled. At the same time, a mirror is used to reduce the projection distance and thus reduces the footprint of the Virtual Wall into half. Five major virtual reality hardware are integrated into the Virtual Wall, namely, 6-degree-of-freedom (6-DOF) tracker system, non-depolarized projection screen, polarized filter, polarized glasses and active-stereo glasses with emitter. The magnetic-type sensor tracker (Polhemus Patriot) is used in this research, which consists of a system electronics unit, a transmitter, a receiver (for head tracking) and a digitizer (user's manipulator). The maximum distance of the sensor tracker at which the resolution and noise performance of the system can be realized is 900 mm at default settings. Even though the useful range can be extended up to 1520 mm, this causes reduction in the accuracy of the data. These hardware are used to visualize and manipulate the virtual objects created in the virtual environment. The ergonomic dimensions (visual and arm reach) of the Virtual Wall needs to be decided. In this research, the visual limit can be neglected for the following reasons:

- Visual position limits: The user can simply walk to the position of his/her choice within the range of the sensor. This means that the user can walk to the left, right, near to or far from the screen if he/she intends to see the virtual objects clearly.
- Visual orientation limits: The 6-DOF sensor is attached to the glasses and virtual environment which will be updated according to his/her viewing angle for all attitudes.

The arm reach is the primary concern for screen position. The horizontal position has no effects on the arm reach since the user can walk either to the left or right if he/she is unable to reach the virtual objects. The vertical position of the projection screen is calculated based on the standing position of the user. The anthropometrics data for the Malaysian population are considered since the system is designed for a manufacturing environment [34]. The vertical position is designed to accommodate 95% of the population from short users (maximum height) to tall users (minimum height) in standing position. The height dimension is within the range of 2.5–97.5 percentiles. The reachable height depends on the shoulder height (*H*), arm reach forward length (*L*) and arm reach angle (*θ*) of the workers. Table 1 shows the parameters for standing position based on the anthropometrics data of Malaysian workers. From Table 1, the minimum reachable height can be calculated using the following equation:
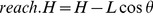
(1)where:
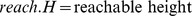


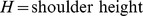


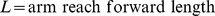


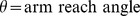



**Table 1:** Parameters for standing position.

Specifications	Male (mm)		Female (mm)	
	Min	Max	Min	Max
Shoulder height, *H*	1,223.00	1,462.56	1,157.00	1,356.96
Arm reach forward length, *L*	724.00	920.00	690.56	843.44
Minimum reachable height – assumed the user's hand is vertically pointed down, with the arm reach angle, *θ* = 30°)	596.00	665.82	558.96	626.52
Maximum reachable height – assumed the user's hand is vertically pointed up, with the arm reach angle, *θ* = 150°)	1,850.00	2,259.30	1,755.04	2,087.40

Therefore, the minimum vertical reachable height is 665.82 mm and the maximum vertical reachable height is 1,755.04 mm. The design accommodates 95% of Malaysian workers.

### Sensor Configuration

**Figure 2:**
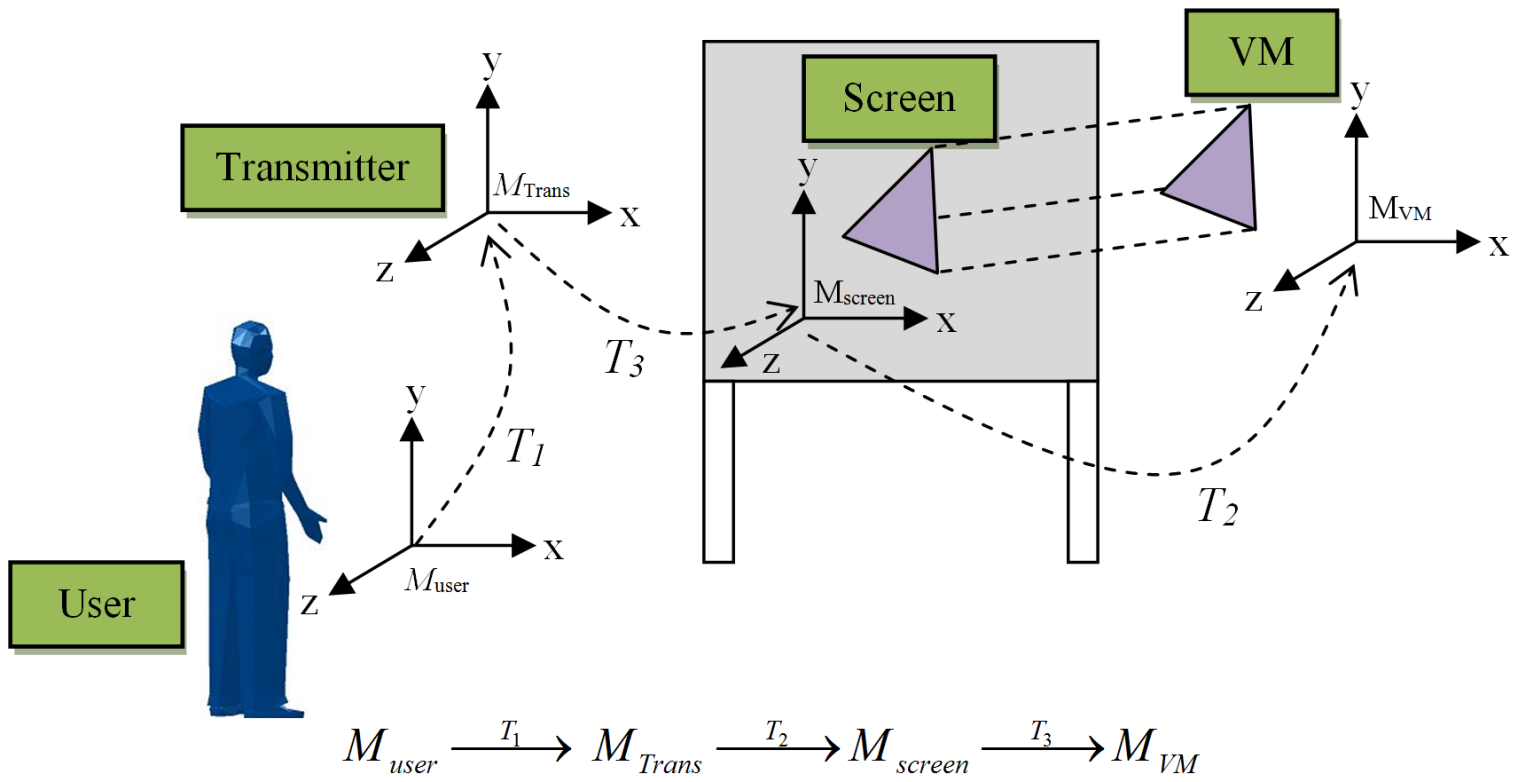
Reference coordinate system for Virtual Wall.

The Virtual Wall used by VR-RoWL involves four coordinate systems, namely, user, sensor transmitter, projection screen and virtual environment (VM). A standard reference coordinate system needs to be selected or defined, whereby other systems can be transformed into this coordinate frame. Thus, the projection screen coordinate system is used as the reference frame, as shown in Figure 2. The transformation between frames can be simplified into the following configurations:

- First, the user's coordinate system needs to be transformed into the transmitter coordinate frame. In this research, the position and orientation of the head and hand are tracked using sensors and the sensors are referred to the transmitter coordinate frame. Furthermore, the default location of the transmitter is programmed at the origin without the rotation angle. Therefore, the transformation matrix *T_1_* can be obtained through the position and orientation of the user's head/hand which is referenced to the transmitter, and these data can be directly tracked by the 6-DOF sensor and digitizer.
- The virtual scene is projected (clipped) onto the screen. The actual dimensions of the screen are used to set up the OpenGL projection, and the virtual objects will appear on a 1-to-1 scale (full-scale), which is the same as the actual size. The centroid of the projection screen is fixed as the origin of the virtual environment. Hence, the transformation matrix *T_2_* represents the position and orientation of the virtual objects referenced to the screen, and these data are referred to the global coordinate system of the virtual environment.

Only two coordinate frames need to be considered, which are transmitter and screen. Both coordinate frames are configured as the origin in their respective frame. Consequently, the transformation matrix *T_3_* can be obtained by measuring the distance and rotation angle of the transmitter which is referenced to the centroid of the screen. The user will feel as though he/she is located within the virtual manufacturing system and uses the same coordinate system through these transformation functions.

**Figure 3:**
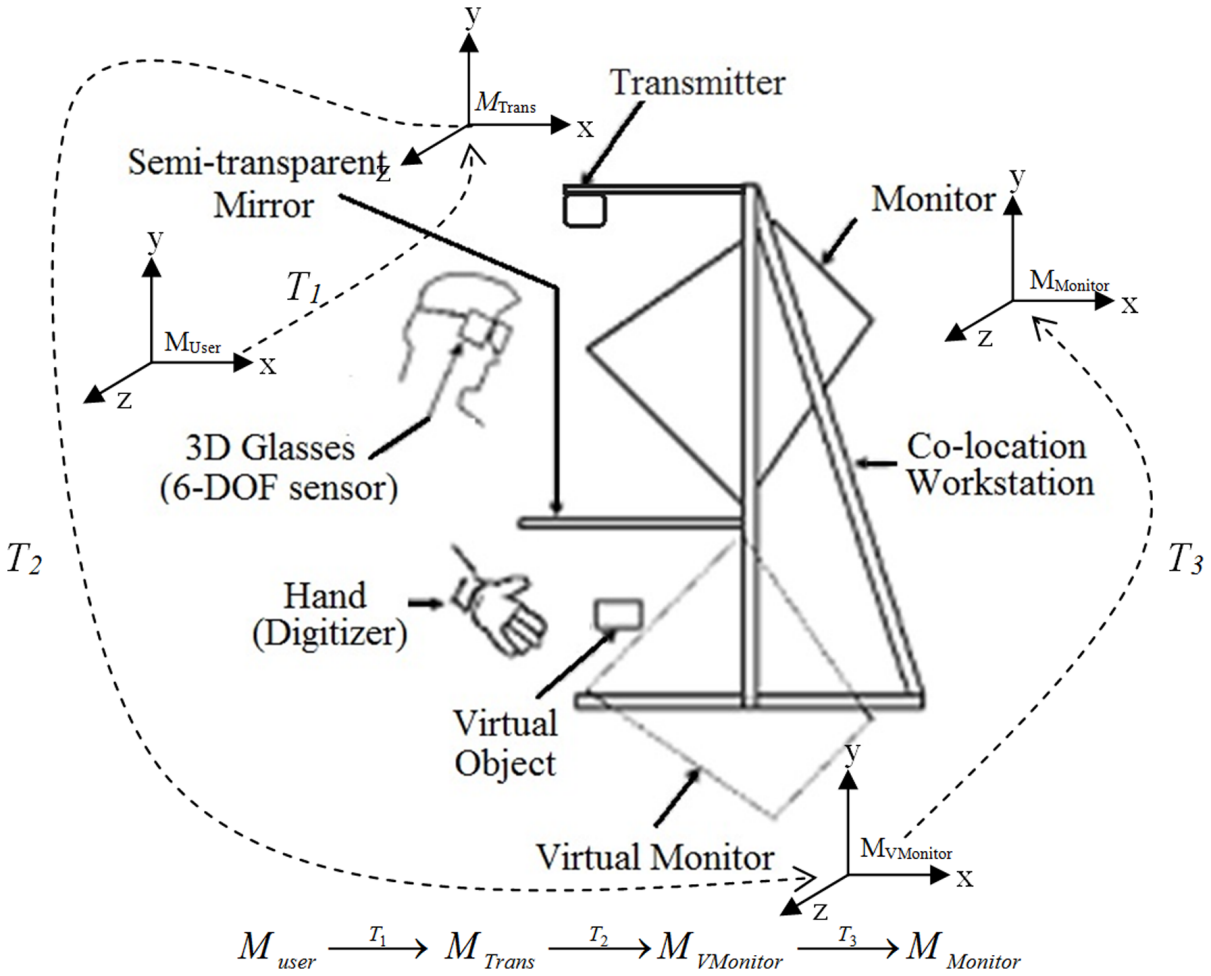
Coordinate system for ErgoVR workstation.

ErgoVR station is another configuration used in the robot teaching system (VR-RoT). The ErgoVR station is designed to make the user feel, see and interact with objects in the most natural and ergonomic position. The user will be able to see and interact with virtual objects in the same place, as shown in Figure 3. Thus, the screen coordinate system can be replaced by a new coordinate system, which is termed as the virtual-monitor coordinate system. The transformation of the frame for the VR-RoT can be simplified into the following configurations:

- Similar to the VR-RoWL condition, the user's coordinate system needs to be transformed into the transmitter coordinate frame. The transformation matrix *T_1_* is obtained directly from the recorded data.
- Based on the unique design of the co-location workstation, the virtual monitor will appear as the reflection of the actual monitor. Therefore, a mirror transformation matrix (*T_3_*) at x-axis is applied to achieve the desired effect.

### Robot Command Generator

The process path generated by the VR-based robot teaching system is recorded in a text file. Following this, the data acquisition and generator are designed to generate the robot commands, in which the generator is programmed specifically for KUKA industrial robots. The command generator is capable of converting the generated path into KUKA readable and executable formats. Hence, the robot commands can be tested by the KUKA OfficeLite simulator before being loaded into the real physical robot.

The software architecture of the KUKA robot system must be identified before conversion between VR-RoT and KUKA robot system can be established. Additionally, KUKA programming language and syntax must be analysed and studied in depth before conversion can be carried out. Each KUKA robot command is formed by two files, i.e. source file (*.src) and data file (*.dat). The data file contains data of the coordinate points whereas the source file consists of robot motion data such as velocity etc.

**Figure 4:**
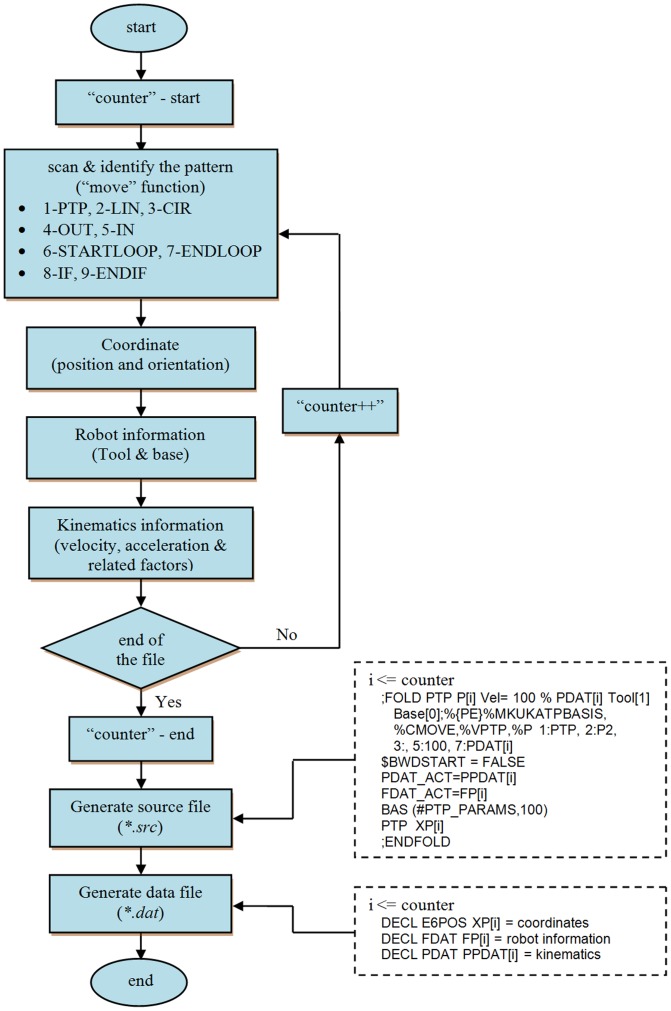
Flow chart of robot command generator.

In this research, the content of each source file is similar to one another, whereby the motion of the robot will be determined during the actual robot testing. However, the source file is somewhat dependent on the data file. For example, the motion loop controls the dynamics and type of movement from one point to another, whereby the number of loops (counter) must tally with the number of points in the data file. The data file consists of all points in the path. Figure 4 shows the flow chart of the robot command generator.

**Figure 5:**
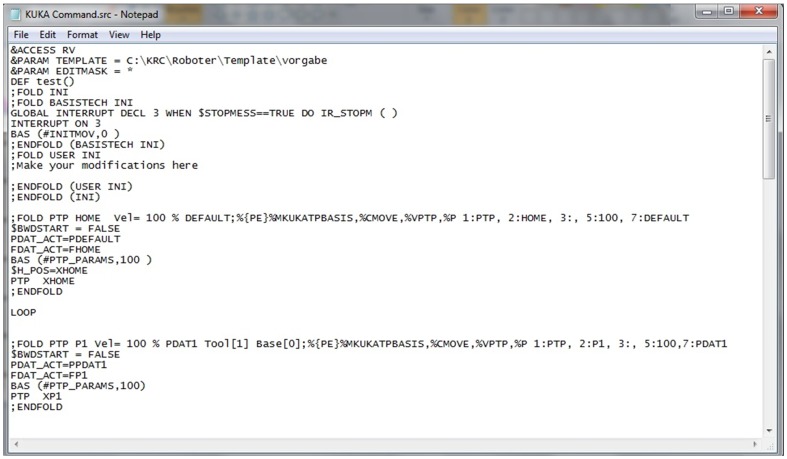
Example of source file (*.src) generated by the VR-based system.

**Figure 6:**
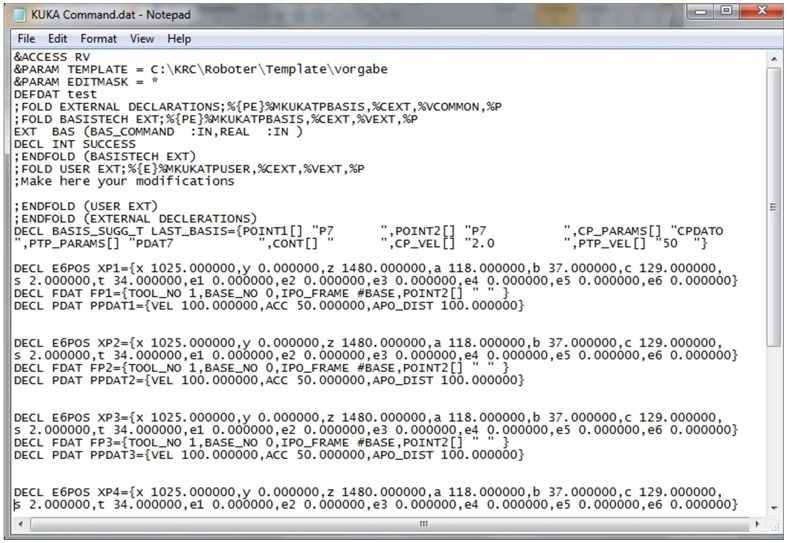
Example of data file (*.dat) generated by the VR-based system.

In general, there are six parameters used to define a point in the KUKA robot command, namely, *x*, *y*, *z*, *pitch*, *yaw* and *roll*. Thus, the coordinates tracked by VR-RoT can be inserted into the data files accordingly. Figure 5 and Figure 6 shows an example of the source file and data file, respectively.

### Software Architecture

**Figure 7:**
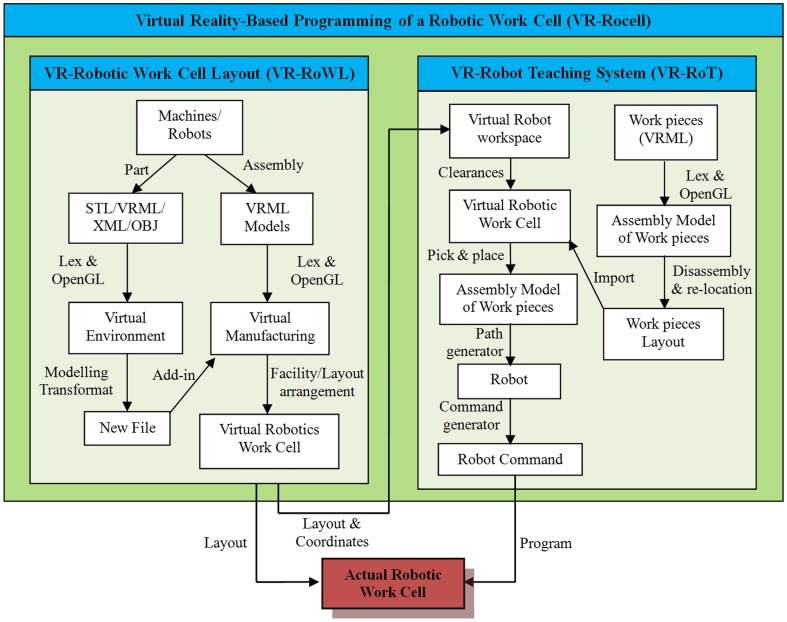
Overview of the project.

The virtual reality-based programming of a robotic work cell (VR-Rocell) is developed by solid modelling using commercial CAD software. The 3D models can be saved in a universal file format, either in STL, VRML, XML or OBJ. Following this, these models are integrated into the VR-Robotic Work Cell Layout (VR-RoWL) using a generic approach, in which the assembly models are saved in the VRML format. Virtual models can be transformed and saved in new files. The assembly models can be designed, modified and re-arranged accordingly. The models will be used to teach the robot path via the VR-based Robot Teaching system (VR-RoT). The robot commands and layout information can be loaded into the actual robotic work cell to execute the desired tasks. In addition, the VM models can be saved in different universal file formats. Therefore, the output models from one VM can be transferred or imported to another VM in the universal file format. Figure 7 shows the overview of the research.

### 3D Models and Coordinate System in VR-RoWL

In the robot layout planning system (VR-RoWL), the layout is re-arranged according to the user's input (via digitizer) in the VRML format. In this case, the models inside the assembly file solely consist of 3-DOF, which allows translation in the horizontal plane (*xz*-plane) and rotation around the *y*-axis. This is due to the constraints of the physical (real) environment, such that the models must be placed on the floor with the *y*-axis always equal to zero. Thus, the machines/robots are unable to rotate around the *x*- and *z*-axes.

The global coordinate system in the virtual environment is the fixed reference coordinate system for all virtual objects. The global coordinate system maintains the position and orientation of the models such that they are unaffected by modelling transformation. The coordinate system is located on the virtual floor rather than on any virtual object since the virtual objects are allowed to move and hence, only the coordinates of the transformed models will be updated. The relative distance between the virtual objects can be calculated using vector analysis. As an example, the material handling of a milling machine is handled by an industrial robot while an operator is assigned to the lathe machine. The new layout involves switching the role (location) of the robot and operator. The global coordinate is fixed at the front-centre of the grid and the transformed coordinates (set-A) of the virtual objects are tabulated in Table 2. An additional set of coordinates (set-B) is shown if the industrial robot is selected as the reference frame (local coordinate) for comparison purposes.

**Table 2:** Global coordinate versus local coordinate in layout design.

	Virtual Object	Original Layout	New Layout
**Set A**	Forklift	(−900, 0, 400), 135°CCW	(−900, 0, 400), 135°CCW
	Milling	(−200, 0, 1500)	(−200, 0, 1500)
	Lathe	(1000, 0, 1000), 315°CCW	(1000, 0, 1000), 315°CCW
	Robot	(−200, 0, 1000), 270°CCW	***(550, 0, 650), 45°CCW***
	Operator	(550, 0, 650), 135°CCW	***(−200, 0, 1000), 180°CCW***
**Set B**	Forklift	(600, 0, −700), 225°CCW	***(−120.2, 0, 848.5), 90°CCW***
	Milling	(−500, 0, 0), 90°CCW	***(70.7, 0, 1131.4), 315°CCW***
	Lathe	(0, 0, 1200), 45°CCW	***(565.7, 0, −70.7), 270°CCW***
	Robot	(0, 0, 0)	***(0, 0, 0)***
	Operator	(350, 0, 750), 225°CCW	***(−282.8, 0, 777.8), 135°CCW***

In general, the coordinates of the virtual objects in set-A can be measured directly from the reference point and are independent of each other. Therefore, only the position and orientation of the moving objects (operator and robot) are updated whereas the others are maintained at the same position. In set-B, the robot (local coordinate) is selected as the reference frame, and it is moved and rotated. Consequently, the reference frame changes even though the coordinate of the robot always remains at (0, 0, 0). Hence, the coordinates for other objects need to be re-calculated based on the new position and orientation of the industrial robot. For this reason, set-A is more suitable and practical for use in a flexible manufacturing environment. The global coordinate system is then applied in the VR-RoWL.

### 3D Models and Coordinate System in VR-RoT

The assembly format is also used in the robot path teaching system. Before proceeding to the path teaching process, the user is required to plan the robot assembly process in advance, which is based on the reverse engineering concept. Firstly, the original assembly model of the workpieces is imported into the virtual environment at the output buffer. Following this, the user has to disassemble the imported assembly models and place the related parts in the assembly area. Finally, the user is required to move the components into the input buffer accordingly. Hence, validation of the assembly models can be carried out in the assembly area and input buffer. Only then the user can demonstrate the assembly process in the virtual environment and this assembly process will be recorded as the robot path.

The imported model is validated, and the vertices and triangle meshing data are checked and compared. The coordinates of the vertices and triangle meshing sequence are also confirmed. Validation of the disassembled model in the assembly area is carried out (Table 3). The imported assembly models are then dismantled and placed in the assembly area accordingly. The layout of the components is used to design the assembly jig and the VR-based robot path teaching system. Finally, the components are moved into the pallet at the input buffer and the output model is validated accordingly.

**Table 3:** Example of validation in VR-RoT.

Parameter		Original (Solidworks)	Imported (VR-RoT)	Dismantled (VR-RoT)	Exported (Internet browser)
Geometric Information	Number of components	3	3	3	3
	Total vertices	3708	3708	3708	3708
	Total triangles	1236	1236	1236	1236
Vertices' coordinates checking		Yes (same)	Yes (same)	Yes (same)	Yes (same)
Triangle meshing sequence checking		Yes (All CCW)	Yes (All CCW)	Yes (All CCW)	Yes (All CCW)

The origin of the industrial robot coordinate frame is located at its base. However, the local coordinate system is a convenient and common method of robot programming in a virtual environment, and is located at the end effector of the robot arm at the “home” position. If the user likes to change the end effector, the relative distance between the new end effector and work-pieces need to be updated although the origin (robot base) is unaltered. Therefore, the local coordinate system is preferable for use in VR-RoT.

### Frame Delay

Real-time updating simulation is an important visual feedback in order to provide a sense of presence in the virtual environment. Moreover, simulation delays will create flickering images, which will cause cyber sickness. In general, delays are caused by insufficient computational load for handling large quantities of polygons in the virtual environment. The situation may become more critical if the user likes to interact with virtual objects in real-time. Furthermore, the active stereo is set at 120 Hz (120 frames-per-second) to create flicker-free stereoscopic images. Therefore, frame delays should be validated by checking the refreshing rate (frames-per-second) in real-time simulations. The validation process is checked with examples of virtual environment and the results are tabulated in Table 4.

**Table 4:** Validation of frame delay.

Virtual environment	Passive stereo (60 Hz)		Active stereo (120 Hz)	
	without sensor	with sensor	without sensor	with sensor
Five (5) Equipment with Vertices (21,040) and Triangles (38,145)	√	√	√^*^	√^*+^
Five (5) Equipment with Vertices (54,107) and Triangles (97,275)	√	√	√^*^	√^*+^
Nine (9) Equipment with Vertices (140,116) and Triangles (142,608)	√	√	√^*^	√^*+^

The validation results show that the virtual environment created matches the computational load for the selected computer workstation. There are no frame delays when the refreshing rate is tested at 60 frames-per-second (FPS or Hz). The first two frames fall below the rated FPS (marked by “*” in Table 4) when the refreshing rate is doubled. This is attributed to the initial processing of the rendering process, whereby the OpenGL technique “Display List” is used for compiling for later use. This technique evaluates all rendering information (vertices and triangles) and stores the information into the server machine at the beginning of the process. The information created can be re-used without re-evaluating and re-transmitting the data during simulations.

Furthermore, the frame delay remains constant (120 Hz) when the sensors are activated in the active stereo mode. However, the frequencies of the sensors are limited to 60 Hz. Thus, the following methods are used to overcome frame delay:

- The following frame will be updated accordingly when the data is triggered and sent from the sensors. A total of 60 frames will be updated based on the input information from the sensors in an event-mode input model.
- The previous information will be used to update the frames if no data is received. The information is applied to the remaining frames, where the OpenGL is able to simulate the virtual environment until the next input is received (marked by “+” in Table 4).

## Results and Discussion

A case study is conducted at the Robotics Laboratory, Department of Mechanical Engineering, Faculty of Engineering, University of Malaya. The industrial robotic work cell is required to assemble an electronics casing which consists of three components: a main casing and two identical heat sink assemblies. The main casing is made of aluminium and the heat sink assemblies are attached onto the main casing. Each heat sink assembly is a combination of a heat sink and a side plate, which has been assembled prior to the robotic work cell. The work cell initially consists of two industrial robots (KUKA KR6/2). However, due to the high maintenance and performance of the robots (payload: 6 kg only), the existing robots are replaced with KUKA KR16 KS (payload: 16 kg). Therefore, a new layout design is required since both robots have different workspace and reach requirements. The machines and robots are listed in Table 5.

**Table 5:** Industrial robotic work cell.

No.	Machine/Robot	Task
1	KUKA KR 16 (A)	Pick-and-place
2	KUKA KR 16 (B)	Fastening
3	Assembly jig with pneumatic system	Assembly area for the casing
4	PLCs	Synchronize and control assembly process
5	Pallet with fibre optic sensor	Input buffer and use sensor to trigger process

**Figure 8:**
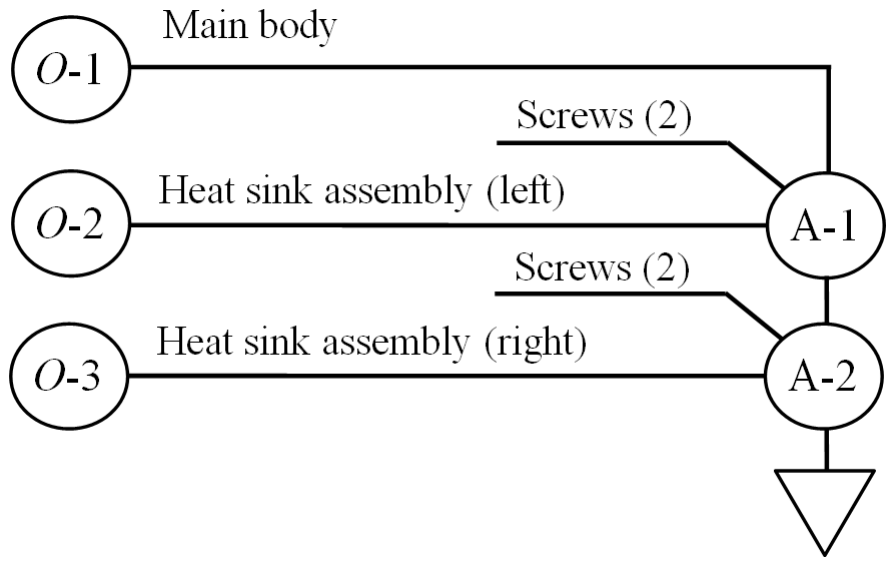
Assembly process chart for case study.

The assembly process of the electronics casing is identified during the design stage, as shown in Figure 8. Firstly, the main body (*O*-1) is placed at its position in the jig. Following this, the left heat sink assembly (*O*-2) is fixed onto the main body using two screws. Finally, the right heat sink assembly (*O*-2) is fixed onto the main body using two other screws.

### VR-Robotic Work Cell Layout (VR-RoWL)

The 3D models of the robots and related components are developed using commercial CAD modelling software. Following this, these models are imported into the virtual work cell for layout planning, in which the assembly model is saved in the VRML format. The layout of the laboratory needs to be studied in advance. The location of the power supply, entrance (door), windows (if any), materials supply and others should be confirmed before re-arranging the layout of the robotic work cell. In this case study, the upper-right corner of the laboratory is assigned as the global coordinate system. The dimensions of the robotic zone will be used to set up the layout for the VR-RoWL. All components and machines must be located within this zone, with the exception of materials supply mechanisms/machines.

**Figure 9:**
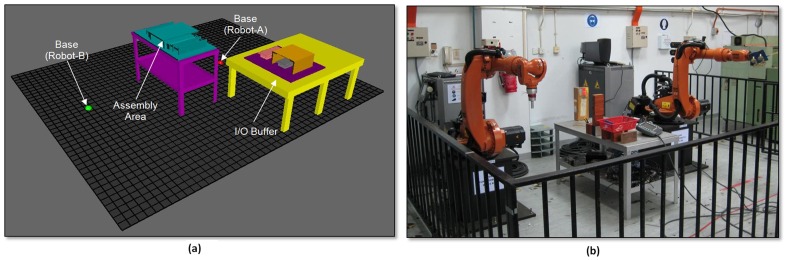
(a) Proposed robotic work cell (b) Actual robotic work cell setup.

The original assembly model is then imported into the VR-RoWL. The models are free to move within the fixed zone (grid). In general, the robots should be placed in parallel and facing towards the positive *y*-axis direction. The assembly zone is located between the robots and within the workspace of both robots. This layout design can be implemented for various assembly processes involving two robots, whereby the materials supply is located in front of the robots. Therefore, the new layout can be obtained by moving the related machines/robots in the virtual layout. The new layout can be saved in the VRML format, which can be shared between various Internet browsers. The proposed robotic work cell created by the VR-RoWL and the actual laboratory setup are shown in Figure 9.

The laboratory is set up according to the above mentioned criteria and the proposed new layout design. The actual dimensions of the equipment are measured based on the proposed global coordinate system. Comparisons between the actual coordinates and virtual layout coordinates for the robotic work cell are tabulated in Table 6. There are some constraints while installing the robots and equipment in the actual laboratory setup; one of them being the accuracy of the measurement tools. Thus, the unit used in the actual setup is centimetres (cm) and the tolerance is ±0.5 cm. The orientation of the equipment in the actual installation is difficult to control and therefore the tolerance is set as ± 3° (for all axes). The digital coordinates (virtual layout) do not impose limitations on the environmental conditions (e.g. floor) and instrumentations (e.g. measuring tape).

**Table 6:** Comparisons between virtual layout and laboratory setup.

No	Equipments	Virtual Layout (x, y) (mm)	Laboratory (x, y) (mm)	Absolute Variation (x, y) (mm)
1	KUKA KR 16 (A)	(1459, 1716)	(1450, 1700)	(9, 12)
2	KUKA KR 16 (B)	(3965, 1713)	(3950, 1700)	(15, 13)
3	Input buffer (Conveyor)	(1556, 3203)	(1570, 3230)	(14, 27)
4	Output buffer (Conveyor)	(1555, 3209)	(1570, 3230)	(15, 21)
5	Assembly jig (Table)	(2515, 2041)	(2530, 2025)	(15, 16)

There are differences between the virtual layout and actual laboratory setup, and the virtual layout provides an overview of the arrangement. There are no measurement tools used in the virtual environment. The user locates the equipment using his/her visual feedback only. For example, the user is unable to “see” a difference of 5 mm in a virtual environment. The user is also unable to “see” whether two robots are exactly located at the same axis (*y* = 1700 mm). This can be improved by applying the “snap-fit” feature, in which each grid is 10 mm.

### VR-Robot Teaching System (VR-RoT)

The imported assembly model is located on the input buffer by default. The user is requested to dismantle the assembly model and move the components to the pallet and arrange them accordingly. In this mode, only the final position and orientation of the components will be stored, neglecting the path of the movement. The path planning mode is then activated after assembly planning and the user is requested to either plan the robot path by moving the components (material handling, e.g. suction cup) or demonstrate the process path (material processing, e.g. fastening). The path will be recorded and translated into robot commands. The loaded information will not be substituted by the final location of the components. In addition, there are two basic ways of describing the movement of industrial robots in a robot control system, i.e. angle of the manipulator's joints or location of the end effector. If all joint variables are known, then forward kinematics can be used to determine the position and orientation of the robot's end (tool centre point). However, inverse kinematics is used to calculate the value of each joint variable by knowing the position and orientation of the end effector. Thus, the inverse kinematic model is developed and used in this research [28].

**Figure 10:**
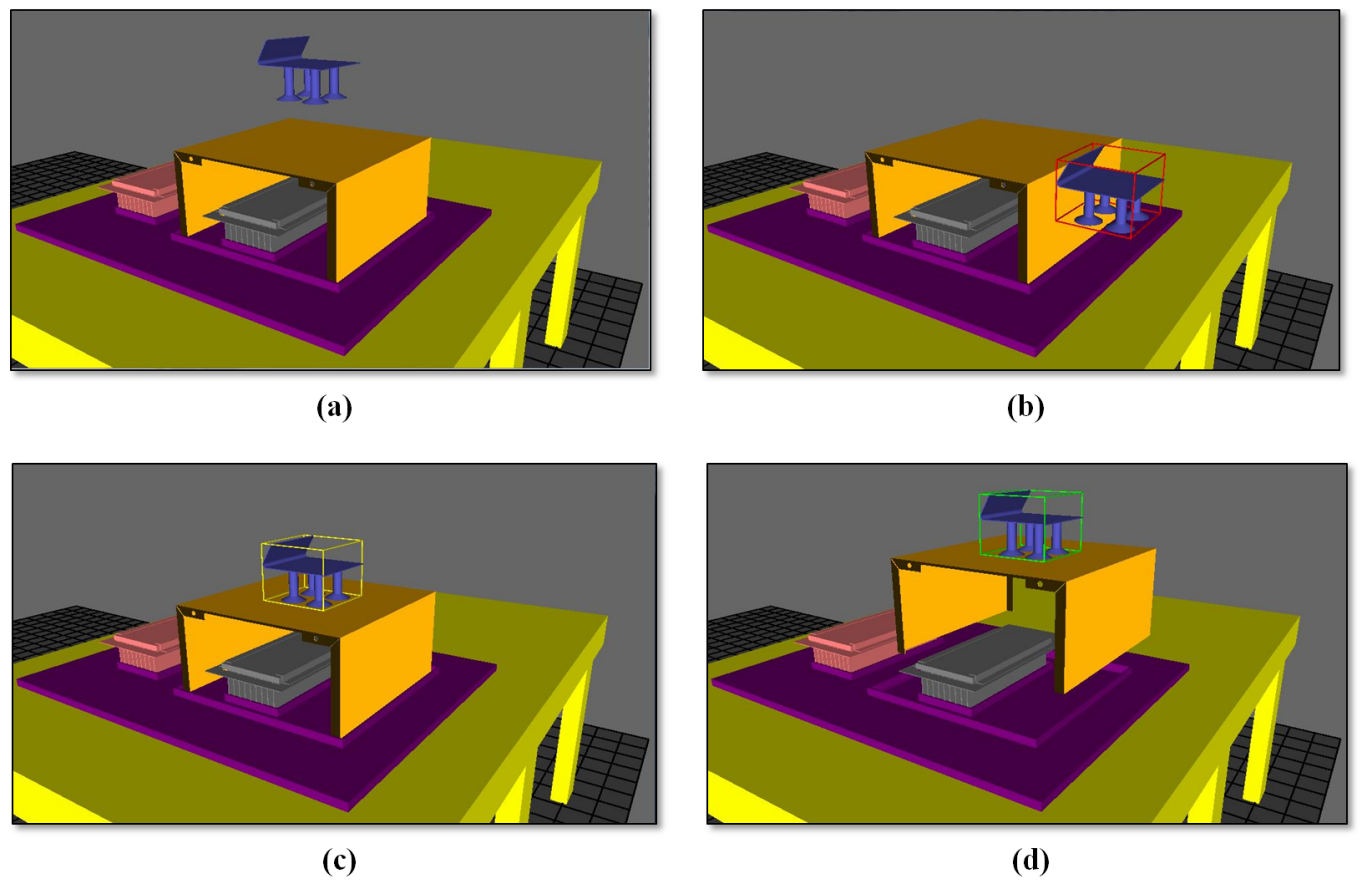
Bounding box for end effector.

Robot-A is programmed to pick-and-place (PnP) the components between the input buffer and assembly area. There is no force or tactile feedback provided to the developed robot teaching system. The collision detection algorithms are applied to detect whether the end effector touches the components, in which a bounding box will be drawn at the end effector when collision is detected. Furthermore, the colour of the bounding box will change at different stages of the PnP process, as follows:

- No bounding box: No collision is detected (Figure 10 (a)).
- Red (blinking): Collision is detected between the end effector and workpiece, but the PnP process cannot be continued (Figure 10 (b)).
- Yellow (blinking): Collision is detected between the end effector and workpiece, and ready for the PnP process (Figure 10 (c)).
- Green (blinking): The colour of the workpiece will stop blinking when the end effector is activated. The workpiece is now grasped by the end effector and moves together with it (Figure 10 (d)).

In the virtual teaching system, collision detection is enabled when the end effector touches the components or the components collide with the assembly jig. Thus, the end effector always pick-and-place at the right height (*z*-axis). Only the horizontal positions (*x*-axis and *y*-axis) may differ slightly from the exact location, as shown in Table 7.

**Table 7:** Data of PnP process for Robot-A.

Step	Task	VR generated Coordinate (x, y, z) (mm)	Predefined Coordinate (x, y, z) (mm)	Absolute Variation (x, y, z) (mm)
a1	Pick: Main Body	(1677, −126, 867)	(1670, −120, 867)	(7, 6, 0)
a2	Place: Main Body	(327, −1076, 1200)	(325, −1080, 1200)	(2, 4, 0)
b1	Pick: Heat Sink Assembly (left)	(1283, −115, 750.5)	(1290, −120, 750.5)	(7, 5, 0)
b2	Place: Heat Sink Assembly (left)	(−91, −1078, 994)	(−94, −1080, 994)	(3, 2, 0)
c1	Pick: Heat Sink Assembly (right)	(1635, −116, 750.5)	(1640, −120, 750.5)	(5, 4, 0)
c2	Place: Heat Sink Assembly (right)	(740, −1076, 994)	(744, −1080, 994)	(4, 4, 0)

**Figure 11:**
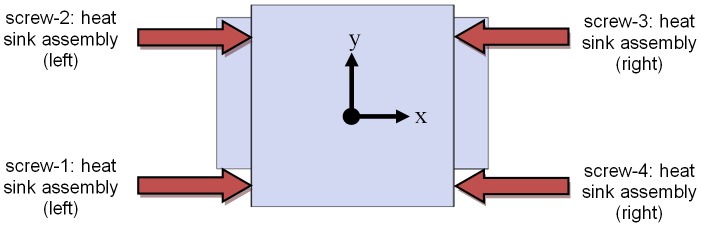
Fastening sequence.

On the other hand, the fastening paths and coordinates need to be controlled, as the fastening coordinates are fixed. These coordinates can be tracked by the screw holes available in the heat sink assembly, which enables the user to change the sequence of the fastening process. Figure 11 shows the fastening sequence defined in the virtual environment and Table 8 shows the coordinates of the fastening process.

**Table 8:** Data of fastening process for Robot-B.

Screw	Predefined Coordinate (x, y, z) (mm)	Fastening direction (Robot's Coordinate system)	Fastening Depth (mm)
1	(113.4, 1270, 1190)	positive *x*-axis	10
2	(113.4, 1570, 1190)	positive *x*-axis	10
3	(536.6, 1570, 1190)	negative *x*-axis	10
4	(536.6, 1270,1190)	negative *x*-axis	10

### Laboratory Testing

**Figure 12:**
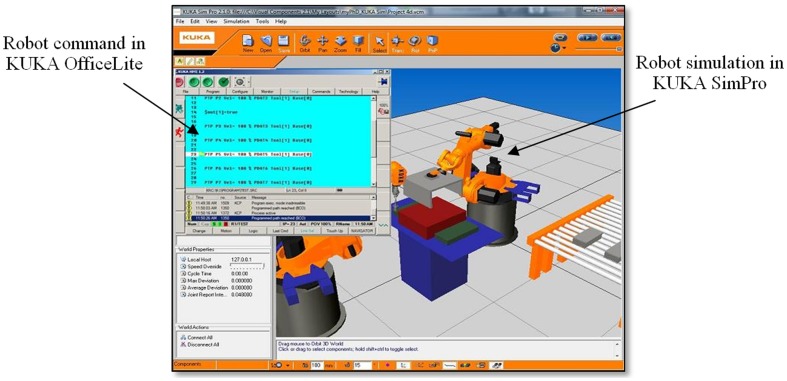
Simulation and validation using KUKA software.

The actual testing is carried out in the Robotics Laboratory. The original work cell consists of two industrial robots of KUKA KR6/2 (payload: 6 kg). The existing robots require higher maintenance and the low payload is unable to fulfil the current requirements. Therefore, the existing robots are replaced with KUKA KR16 KS (payload: 16 kg). Thus, a new layout design is needed since both robots have different workspace, reach, speed and other requirements. Following this, the VR-RoWL model is used to determine and evaluate the layout prior to the actual hardware installation. The robot teaching system (VR-RoT) is used to plan and determine the robot path in advance. The robot process path is taught and the robot commands are generated offline. The layout and process path planned in the VR-RoWL are validated using KUKA SimPro Simulation environment to determine clearance, reach, safety etc. KUKA OfficeLite is used to validate the robot commands generated by the VR-RoT. Figure 12 shows the simulation and validation using KUKA software before being loaded into the actual system.

Finally, the actual robots and all equipment are installed at their respective locations based on the layout generated by the VR-system. The KUKA robot commands generated by the VR-based system are saved into a floppy disk and transferred into Robot-A (pick-and-place) and Robot-B (fastening), respectively. The end effector is then attached to each robot. The assembly jig is assembled with a pneumatic system and the input buffer is equipped with a fibre optic sensor. The PLCs are programmed to synchronize and control the assembly process for all equipment.

The robots are operated at 10% of their maximum speed under two conditions (i.e. with and without load) during laboratory testing. The speed is then increased to 30 and 50% of the maximum speed for both conditions. These changes are made using the control panel of the KUKA robot. The average cycle time of the test (five tests for each condition and speed) is recorded and tabulated in Table 9.

**Table 9:** Average cycle time for various speeds.

Process	10% of Speed		30% of Speed		50% of Speed	
	with load (sec)	without load (sec)	With load (sec)	without load (sec)	with load (sec)	without load (sec)
1. Trigger the sensor	1.0	1.0	1.0	1.0	-	1.0
2. Robot-A: Pick the components from input buffer and place at assembly area	103.12	102.88	36.56	36.28	-	23.08
3. Position the jig	2.0	2.0	2.0	2.0	-	2.0
4. Robot-B: Fastening the four (4) screws.	49.38	49.12	17.41	17.14	-	11.08
5. Position the jig	2.0	2.0	2.0	2.0	-	2.0
6. Robot-A: Pick the completed assembly at assembly jig and place at output buffer. Return to home position.	38.56	38.36	14.16	13.92	-	8.68
**Cycle Time**	**196.06**	**195.36**	**73.13**	**72.34**	**-**	**47.84**

Overall, it can be seen that the cycle time is reduced when the speed of the robots is increased, whereby the relationship between the cycle time and percentage speed of the robot is fairly linear. It is also found that the cycle time without load is slightly faster than that with load, which is about 0.70 and 0.79 sec for 10% and 30% speed, respectively. The suction cup is unable to hold the components firmly when the robot arm travels at high speed (50% speed) and thus the data for 50% speed is unsuitable for load-carrying use in this case study.

## Conclusions

A virtual reality based programming for an industrial robotic work cell has been developed in this research, and is given the name “VR-Rocell”. The system is divided into two major sub-systems, namely, VR-Robotic Work Cell Layout (VR-RoWL) and VR-Robot Teaching System (VR-RoT). The system is designed to improve the human-machine interface (human-robot interface), whereby off-line programming is used to generate robot commands which will reduce robot downtime and prevents the programmer from exposure to potential hazardous environment.

The system has been validated and tested using a case study. Several settings and assumptions have been made during the validation process. The generic approach of integrating and converting file formats is validated for both part and assembly files and the frame delay has also been tested. It is found that the algorithms and approaches used are able to generate a flicker-free virtual environment for both passive and active stereoscopic visualization systems. Moreover, a case study has been conducted for an electronics casing assembly. The results reveal that there is an absolute variation of 9–15 mm (*x*-axis) and 12–27 mm (*y*-axis) between the virtual layout and actual setup. The virtual path teaching shows an absolute variation of 2–7 mm (*x*-axis) and 2–6 mm (*y*-axis). It is found that the variations in dimensions have no effects on the performance of the robotic work cell. Thus, it is concluded that the VR-Rocell can be used in an industrial robotic work cell based on the accuracy and effectiveness achieved in the case study. Future work can be focused on layout and sequence optimization, analysis of human factors in workstation design and multi-criteria decision making for sustainable manufacturing in order to further improve the proposed system.
